# Interventions for Increasing the Quality of Preventive Care at a Free Clinic

**DOI:** 10.7759/cureus.6562

**Published:** 2020-01-04

**Authors:** Matthew Burger, Marisa S Taddeo, Daniel Hushla, Magdalena Pasarica

**Affiliations:** 1 Medicine, University of Central Florida College of Medicine , Orlando, USA; 2 Medicine, University of Central Florida College of Medicine, Orlando, USA; 3 Family Medicine, University of Central Florida College of Medicine, Orlando, USA

**Keywords:** screening, quality assurance, quality improvement, student-run clinic, preventive care

## Abstract

A quality improvement committee targeted six clinically meaningful measures in order to determine which interventions improved preventative care in a free clinic as well as to qualify for certification as a Patient-Centered Medical Home. Four quality improvement interventions were designed and implemented. The outcomes of these interventions were compared with the national average performance and the Center for Disease Control's Healthy People 2020 goals. All outcomes measured exceeded the national averages, and five out of six outcomes were above the Healthy People 2020 goals. The approach outlined may be used by other free clinics aiming to provide quality preventive care for their patient population and to help obtain Patient-Centered Medical Home certifications.

## Introduction

Preventive care services, including routine screenings and monitoring of chronic conditions, allow for the reduction of mortality and morbidity in at-risk populations and could save more than two million lives annually [1]. The Center for Disease Control (CDC) Healthy People 2020 initiative provides outcome targets to be used as guidance by healthcare institutions and encourages providers to provide a cost-effective solution to the health challenges faced by Americans [2]. Additionally, many payers provide incentives for practices with a commitment to continuous quality improvement and a patient-centered approach to care. This is recognized with a certification process put in place by the National Committee for Quality Assurance for Patient-Centered Medical Homes. It is a model of care that puts patients at the forefront of care and builds relationships between patients and their clinical care teams for improved patient satisfaction and outcomes at decreased costs [3].

Despite these national efforts to encourage preventive care visits, utilization rates are about half the recommended rates [4]. Uninsured low-income patients further underutilize these visits and achieve less prevention measures because they face geographic, social, and financial barriers to care [5]. These patients (8.6% of Americans in 2005) are usually cared for in free or reduced-cost clinics [6]. Therefore, improving prevention measures in a free clinic may significantly impact patient outcomes and cost expenditure.

The purpose was to determine what interventions could improve preventive care performance measurements in a free clinic. The long-term goal was to obtain the highest level of recognition (level III) as a Patient-Centered Medical Home from the National Committee for Quality Assurance. To achieve these, a quality improvement committee was created to design and measure the effect of four quality improvement interventions on six clinically meaningful secondary and tertiary prevention measures recommended by the U.S. Preventive Services Task Force [7]. The outcomes were compared with the national averages and the Healthy People 2020 goals [8].

## Materials and methods

Setting

This study was performed at a free clinic providing comprehensive medical care services to low-income uninsured individuals.

*Study design: *A quality improvement medical committee was formed comprising medical personnel, local quality experts, and other stakeholders (nurses, physicians, administrators, partner hospital representatives). This group met periodically to interpret the quality data and design quality improvement interventions. Six quality measures were selected, with the inclusion criteria set by the U.S. Preventive Services Task Force [7]. Inclusion criteria for tertiary prevention and goals were as follows: age between 18 and 75 years and diagnosis of diabetes (for glucose control defined as glycated hemoglobin [HbA1C] less than 9%, and yearly urine protein and dilated eye exam), and age between 18 and 75 years and hypertension (HTN) (for blood pressure [BP] control defined as BP lower than 140/90 mm Hg). Inclusion criterion for secondary prevention of colon cancer was age between 50 and 75 years (with the goal of yearly fecal occult blood test or sigmoidoscopy every 5 years or colonoscopy every 10 years), and the inclusion criteria for secondary prevention of breast cancer were age between 41 and 69 years and female gender (with the goal of yearly screening mammograms). The Institutional Review Board at the University of Central Florida approved this study (IRB000001138).

Interventions Implemented

Using previously published data [9-12], quality committee members’ input, and an interdisciplinary team approach, four interventions were designed in order to address six quality measures.

Interventions took four forms: (1) patient education, (2) provider education on preventive measures, (3) provider education on correct technique for the patient’s BP measurement, and (4) introduction of electronic medical record (EMR) features to serve as reminders. To increase the screening rates for urine protein, dilated eye exam, and breast and colon cancer screening, two interventions were implemented. (1) Electronic reminders were developed and integrated into the EMRs in a special field called “reminders”. Any patient due for the targeted prevention measures had a reminder attached to his/her chart, which would prompt the physician and nurse to address it during the clinic visit. In addition, the providers and nurses received formal education on the following preventive measures: which patients qualify for them, why they are important, and how to properly use the reminders. (2) Periodic EMR reports on target quality measures were run for patients meeting the inclusion criteria. If they were not completed, the medical director would place the indicated orders in the EMR and the patient received an appointment for lab or imaging study and follow-up clinical visit. Since the breast and colon cancer screening generated the most questions from the patients, additional interventions were added for these two measures. (3) Patients meeting the inclusion criteria were provided with an information sheet describing the significance of the prevention test in English and Spanish after discussing with the provider.

Interventions were designed based on the literature, expert input, and local experience and processes. For instance, in our setting, it was believed that poor diabetes control was due to insufficient monitoring of HbA1C (every three months), which was shown to improve compliance and outcomes. Therefore, interventions 1 and 2 were implemented to assure that patients with diabetes have HbA1C tested every three months. HTN seemed to be not controlled based on the in-clinic measurements, but it was controlled based on home measurements. Therefore, it was hypothesized that patients actually have controlled HTN, but the measurements of BP in the clinic were not performed correctly. Therefore, intervention 4 was utilized to educate the nursing staff on the proper measurement of BP after the proper five minutes rest time.

Data Collection and Analysis

Performance reports were generated by analyzing de-identified EMR reports. All records of patients meeting the including criteria were included in the analysis. For each target, performance was compared using results for one year before and after the intervention with the available national performance averages (2015 Healthcare Effectiveness Data) and Healthy People 2020 using descriptive statistics [8].

## Results

Patient Demographics

Using EMR reports, 134 patients met the inclusion criteria for diabetes secondary and tertiary prevention, 262 for tertiary prevention of HTN, 325 for secondary prevention of colon cancer screening, and 334 for secondary prevention of breast cancer screening. Patient demographics are described in Table 1.

**Table 1:** Demographic Characteristics Patient demographic characteristics are presented as percentages from the total number of patients who were included in the study. Age is represented in years. SD, standard deviation; AA, African-American

	Age (mean±SD)	Gender (men/women)	Race (AA/white/other)	Primary language (English/Spanish/other)
Glucose control	53±10	29%/71%	37%/51%/12%	89%/6%/5%
Urine protein screening	53±10	29%/71%	37%/51%/12%	89%/6%/5%
Eye exam screening	53±10	29%/71%	37%/51%/12%	89%/6%/5%
Blood pressure control	55±9	36%/64%	35%/51%/14%	91%/5%/4%
Colon cancer screening	58±4	30%/70%	29%/57%/14%	91%/5%/4%
Breast cancer screening	54±7	0%/100%	32%/55%/13%	91%/6%/3%

Change in Prevention Measures

There was an observed increase in the percentage of patients with diabetes completing urine protein screening from 87.7% before interventions to 100.0% after interventions (P=non-significant [NS]). Colon cancer screening rates increased from 75.0% before interventions to 76.2% after interventions. Breast cancer screening rates also increased from 93.4% before interventions to 94.0% after interventions (P=NS). However, for the patients with diabetes, there was a small decrease in both glucose control, from 85.0 to 84.7% (P=NS), and annual eye exam rates, from 84.6% to 78.1% (P=NS). Also, more patients with HTN were not at goal from 60.7% to 68.5% (P=NS).

Comparison with the national averages and 2020 goals: The clinic performance post-intervention vs. national average vs. 2020 goal was 84.7% vs. 82.0% vs. 83.8% for HbA1C,100% vs. 33.3% vs. 36.6% for annual urine protein screening, and 78.1% vs. 53.5% vs. 58.7% for annual dilated exam, respectively. In patients with HTN, BP was at goal for 58.5% vs. 43.7% vs. 61.2% post-intervention vs. national average vs. 2020 goal, respectively. Colon cancer screening was performed in 76.2% vs. 62.4% vs. 70.5% and breast cancer screening was performed in 94.0% vs. 71.6% vs. 81.1% of the eligible patients post-intervention vs. national average vs. 2020 goal, respectively.

**Figure 1:**
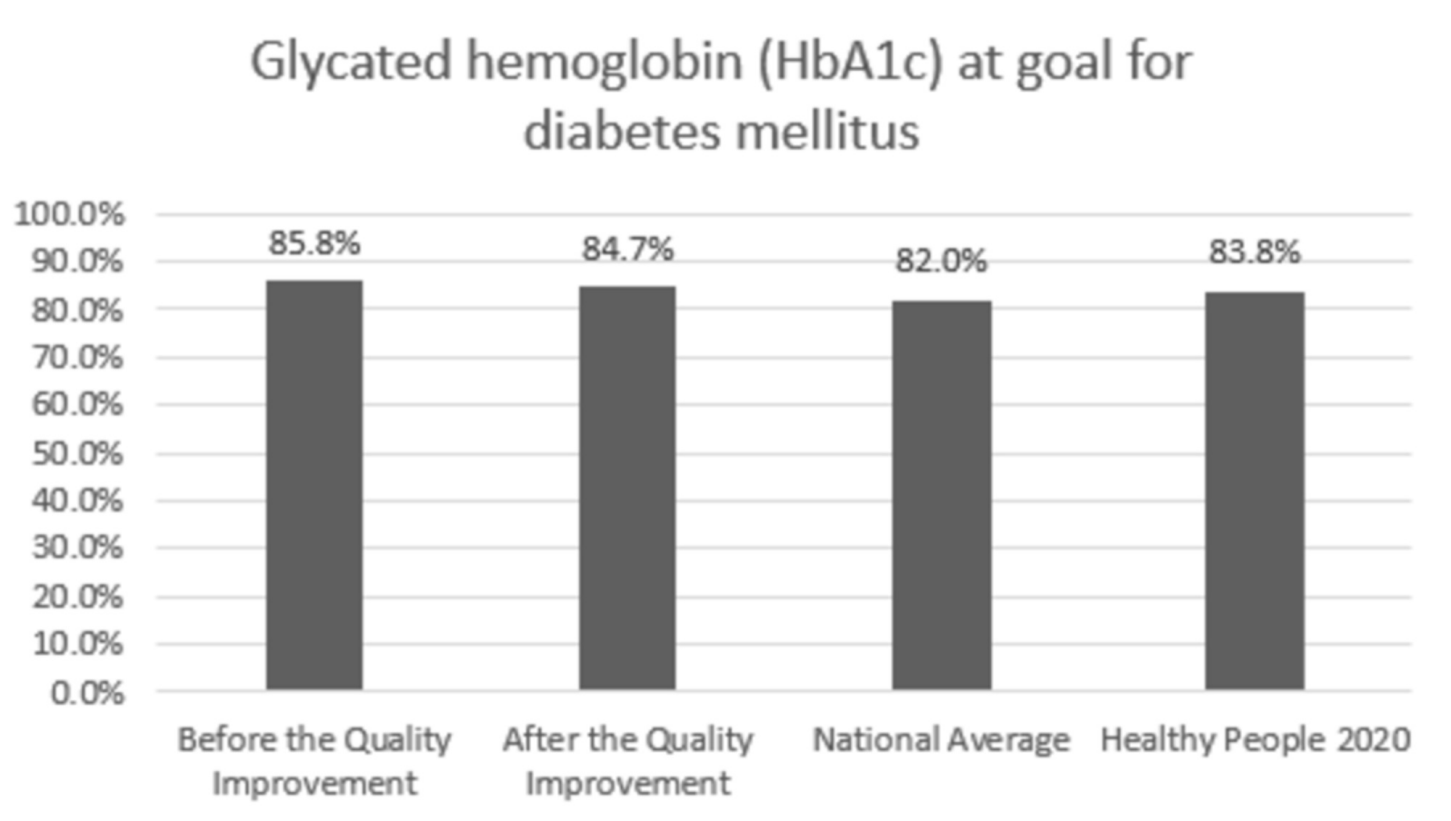
Preventive care performance in blood glucose screening at the a free clinic compared with the national averages and Healthy People 2020 goals

**Figure 2:**
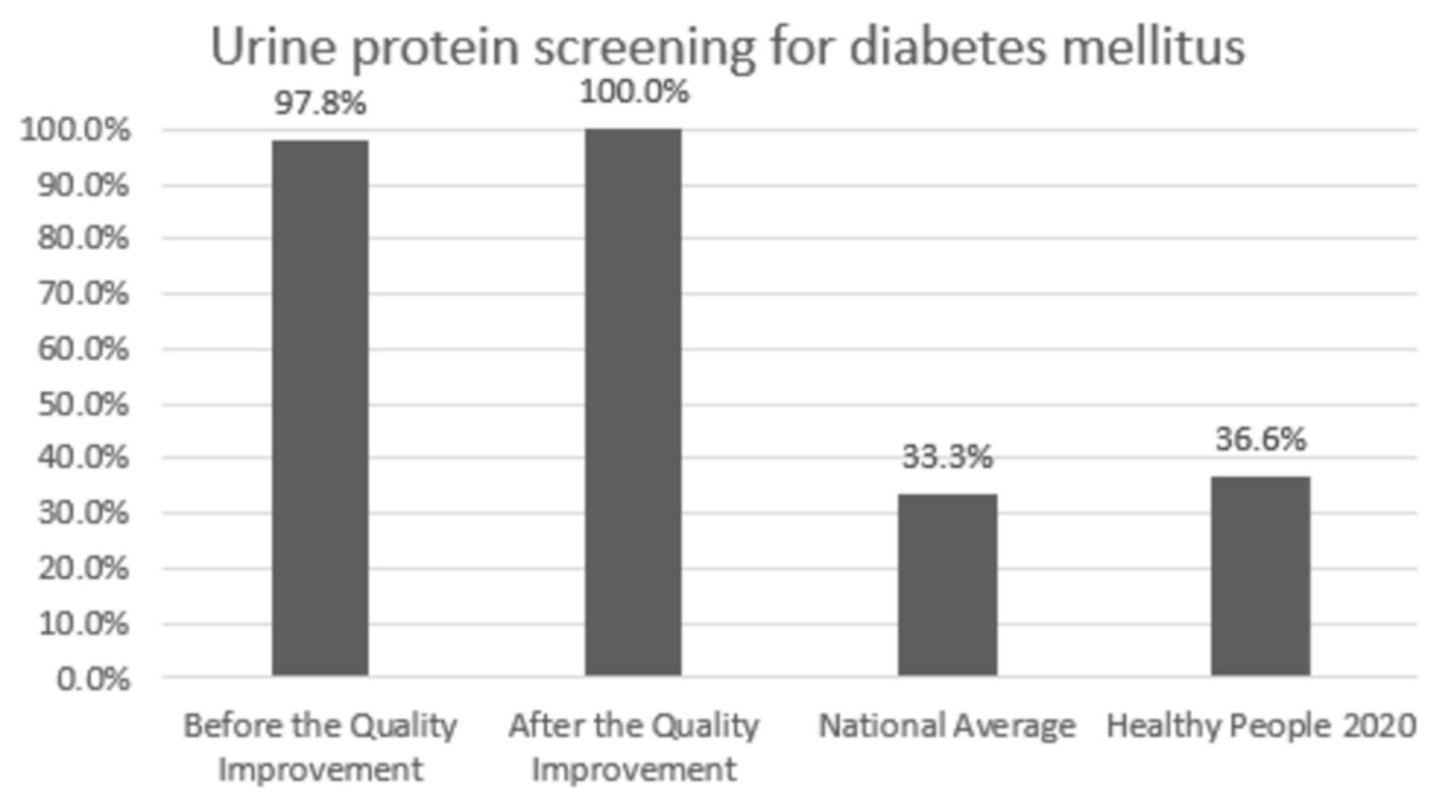
Preventive care performance in urine protein screening at the a free clinic compared with the national averages and Healthy People 2020 goals

**Figure 3:**
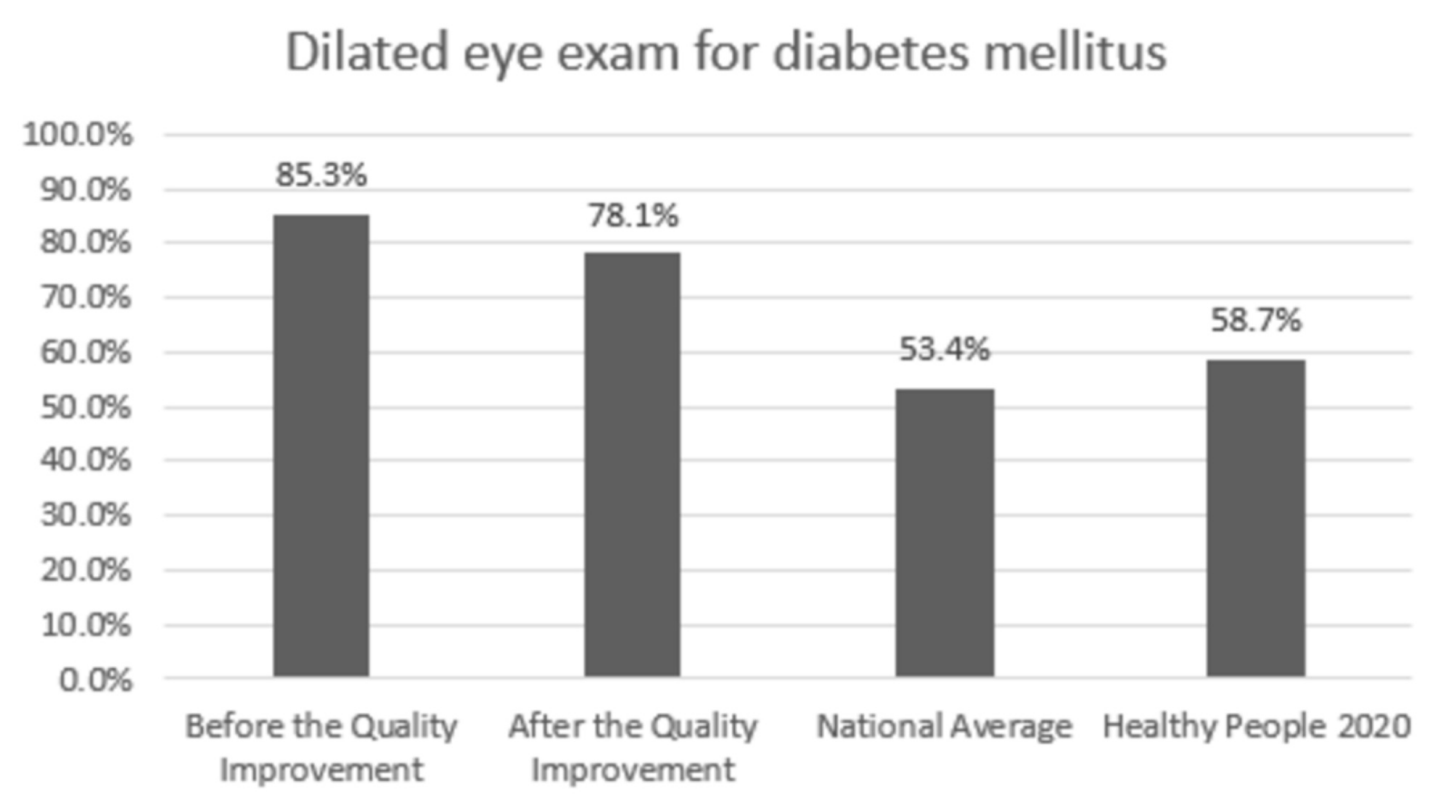
Preventive care performance in eye exam at the a free clinic compared with the national averages and Healthy People 2020 goals

**Figure 4:**
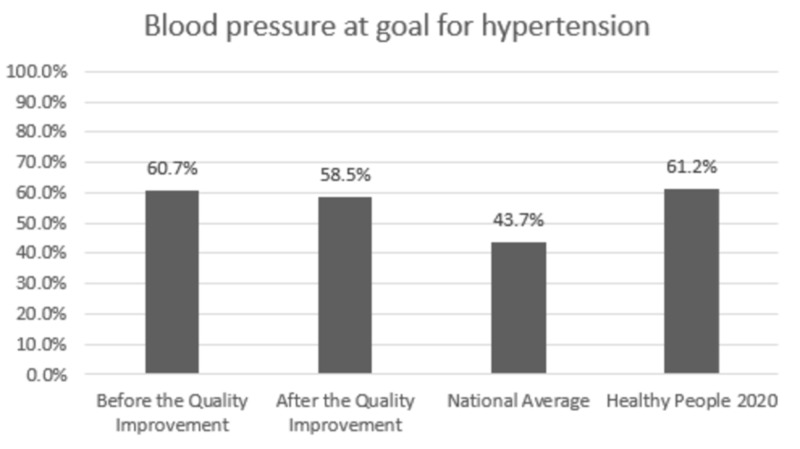
Preventive care performance in blood pressure screening at the a free clinic compared with the national averages and Healthy People 2020 goals

**Figure 5:**
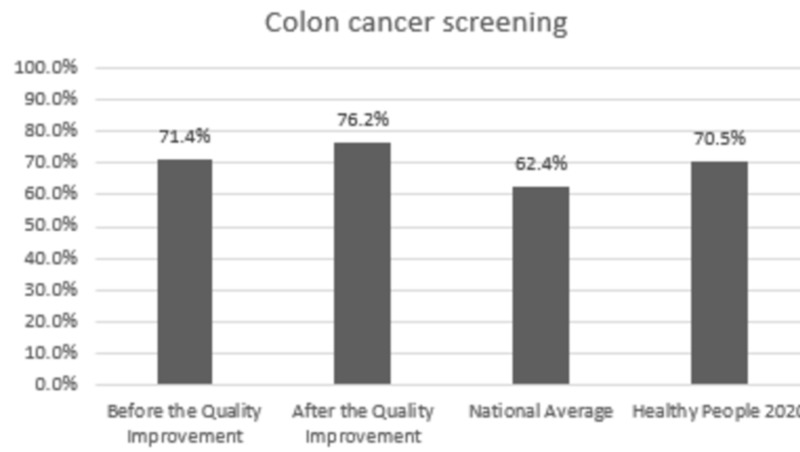
Preventive care performance in colon cancer screening at the a free clinic compared with the national averages and Healthy People 2020 goals

**Figure 6:**
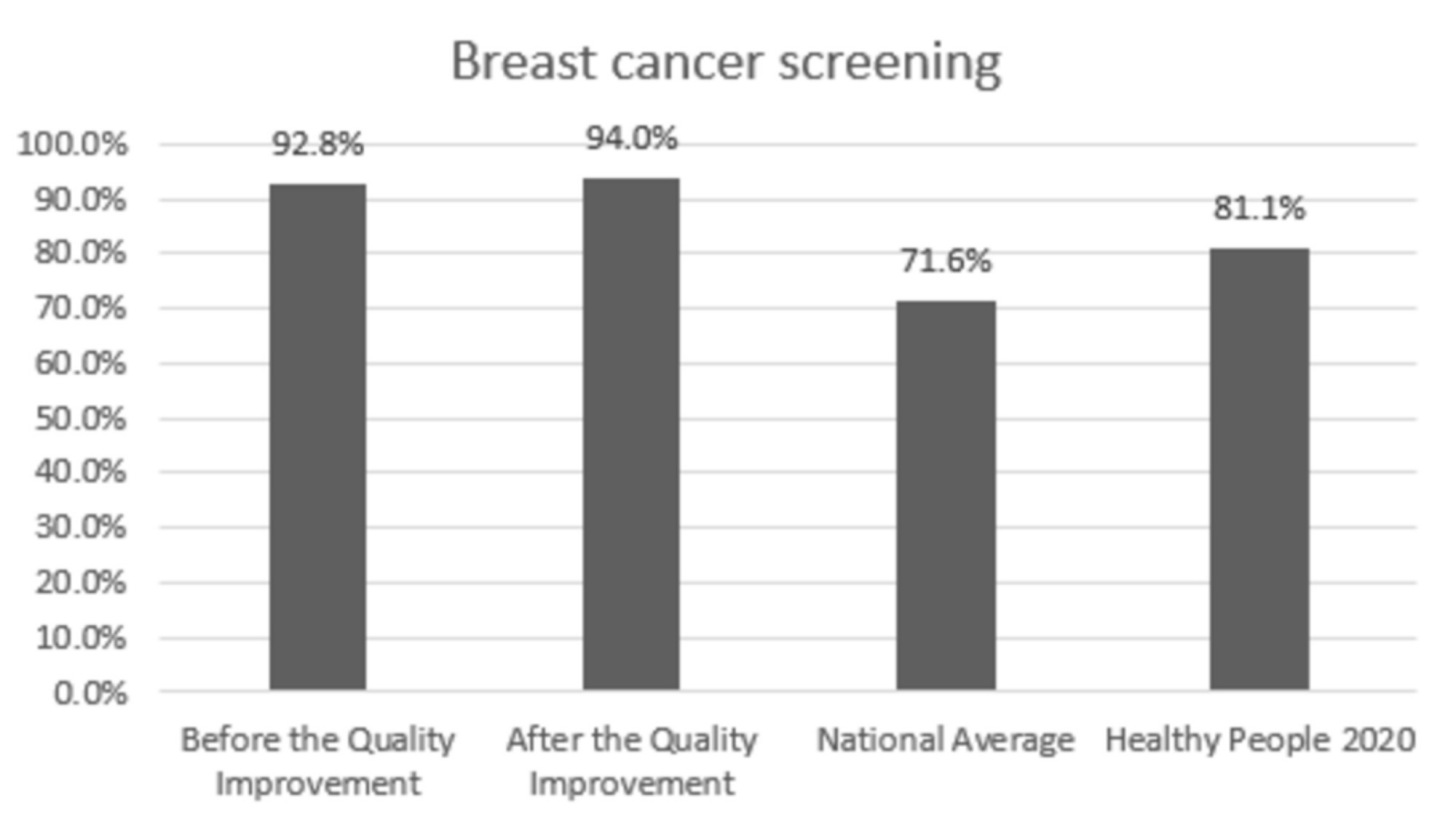
Preventive care performance in breast cancer screening at the a free clinic compared with the national averages and Healthy People 2020 goals

Change in prevention measures and comparison with the national averages and Healthy People 2020 goals are presented in Figures 1-6.

In Figures 1-6, screening rates are reported as percentages of the total population of patients who fit the criteria for screening, and these values are included at the top of each bar. Performance data from the calendar year of 2015 (before the quality improvement projects, which was used as a needs analysis) are represented in the first bar. Performance data after the quality improvement projects were designed and implemented are presented in the second bar. The National average and Healthy People 2020 goals are represented in the third and fourth bars. Figures 1A, 1B, 1C represent measurements for patients with diabetes. Figure 1D represents measurements for patients with HTN. Figures 1E, 1F represent data for cancer screening.

## Discussion

This quality improvement processes in a free clinic contributed to the achievement of the highest level of Patient-Centered Medical Home certification. The four interventions implemented resulted in the improvement of rates for screening of breast and colon cancer in patients at risk and for urine protein screening for patients with diabetes, but they did not improve the control of diabetes, HTN, and yearly dilated eye exam for patients with diabetes. All measurements were higher than the national averages. Importantly, the performance on five preventive measures was above the Healthy People 2020 goals (with the exception of HTN control, where the clinic scored 2.5% lower than the Healthy People 2020 goal).

Results showed that building electronic reminders (intervention 1) and using performance reports to additionally order a screening test (intervention 2) was effective in increasing urine protein screening but not dilated eye exam. This may be due to the protein screening being performed during the clinic visit, whereas the dilated eye exam needed an additional visit with an ophthalmologist. Adding the educational materials (intervention 3), in addition to reminders and performance reports, resulted in improved performance for breast and colon cancer screening, suggesting the application of this approach for other measurements. Future intervention for improving the completion of yearly dilated eye exam should also include providing patients with educational materials in Spanish and English after discussing with the provider.

Interventions did not improve diabetes control or HTN control. This suggests that the hypothesis for the causes of control not at goal (infrequent monitoring of diabetes and improper measurements of BP in the clinic) is incorrect. Maybe the pre-intervention performances were so high that no improvement was possible (the plateau effect). Even with the HTN control performance not being statistically significantly different compared with the Healthy People 2020 goals, we still consider this particular intervention as in need of another cycle of improvement interventions. Future interventions for improvement of diabetes and HTN control should include other interventions previously shown to be effective [13-15], including educational conferences and courses for providers with updates in the management of diabetes and HTN and offering rewards for performance at goal.

Study Limitations and Strengths

The four interventions described were implemented simultaneously, resulting in a limitation to draw specific conclusions about the efficacy of each intervention. The study was also not able to select a certain group of patients for the reports, and therefore some patients included in the post-intervention may have just joined the clinic and did not participate in the interventions. Since this is a free clinic, approximately half of the visits are provided by volunteer nurses and providers, which may influence the rate of consistent implementation of the interventions. This clinic is the site for a student-run clinic, which traditionally has low rates of preventive care performance [16], and their measurements are included in the report. This study is strong because it was implemented at a free clinic, where resources are limited and patients are at high risk of low prevention rate. Therefore, the applicability of the results in such a setting is of high significance.

## Conclusions

Building electronic reminders and using performance reports to additionally order a screening test was effective in improving urine protein screening but not dilated eye exam. Providing educational materials, in addition to reminders and performance reports, resulted in improved performance for breast and colon cancer screening. Increased frequency of diabetes monitoring did not improve diabetes control. Improper measurements of BP in the clinic did not contribute to uncontrolled BP.

Implications

This approach could be used by other free clinics aiming to provide quality of preventive care for their patient population and obtain patient-centered care certifications. The study suggests that free clinics could improve prevention performance using approaches similar to those of this free clinic performing above the Healthy People 2020 goals. This success may be due to high rates of follow-up, which was noted by patients to be the most appreciated feature of the clinic and could be achieved by forming long-lasting relationships with the patients.

This quality improvement process could be used for achieving other goals. In this case, this study created an educational opportunity for a new generation of medical doctors working in the student-run clinic associated with this free clinic. It also created the opportunity for the physicians practicing at the clinic to accomplish their licensing board requirements for quality improvement studies.
